# Vaccine hesitancy and consequences for vaccination coverage in children at 24 months of age, born in 2017-2018, living in the state capitals, Federal District and 12 inner region cities of Brazil

**DOI:** 10.1590/S2237-96222024v33e20231097.especial2.en

**Published:** 2025-01-10

**Authors:** Rita Barradas Barata, Ana Paula França, Ione Aquemi Guibu, Gabriel Munhoz, Carla Magda Allan Santos Domingues, Maria da Gloria Teixeira, José Cássio de Moraes, ICV 2020 Group, Adriana Ilha da Silva, Alberto Novaes Ramos, Ana Paula França, Andrea de Nazaré Marvão Oliveira, Antonio Fernando Boing, Carla Magda Allan Santos Domingues, Consuelo Silva de Oliveira, Ethel Leonor Noia Maciel, Ione Aquemi Guibu, Isabelle Ribeiro Barbosa Mirabal, Jaqueline Caracas Barbosa, Jaqueline Costa Lima, José Cássio de Moraes, Karin Regina Luhm, Karlla Antonieta Amorim Caetano, Luisa Helena de Oliveira Lima, Maria Bernadete de Cerqueira Antunes, Maria da Gloria Teixeira, Maria Denise de Castro Teixeira, Maria Fernanda de Sousa Oliveira Borges, Rejane Christine de Sousa Queiroz, Ricardo Queiroz Gurgel, Rita Barradas Barata, Roberta Nogueira Calandrini de Azevedo, Sandra Maria do Valle Leone de Oliveira, Sheila Araújo Teles, Silvana Granado Nogueira da Gama, Sotero Serrate Mengue, Taynãna César Simões, Valdir Nascimento, Wildo Navegantes de Araújo

**Affiliations:** 1 Faculdade de Ciências Médicas da Santa Casa de São Paulo, Departamento de Saúde Coletiva, São Paulo, SP, Brasil; 2 Organização Pan-Americana da Saúde, Brasília, DF, Brasil; 3 Universidade Federal da Bahia, Instituto de Saúde Coletiva, Salvador, BA, Brasil; Universidade Federal do Espírito Santo, Vitória, ES, Brazil; Universidade Federal do Ceará, Departamento de Saúde Comunitária, Fortaleza, CE, Brazil; Faculdade Ciências Médicas Santa Casa de São Paulo, São Paulo, SP, Brazil; Secretaria de Estado da Saúde do Amapá, Macapá, AP, Brazil; Universidade Federal de Santa Catarina, SC, Brazil; Organização Pan-Americana da Saúde, Brasília, DF, Brazil; Instituto Evandro Chagas, Belém, PA, Brazil; Faculdade de Ciências Médicas Santa Casa de São Paulo, Departamento de Saúde Coletiva, São Paulo, SP, Brazil; Universidade Federal de Mato Grosso, Cuiabá, MT, Brazil; Universidade Federal do Paraná, Curitiba, PR, Brazil; Universidade Federal de Goiás, Goiânia, GO, Brazil; Universidade Federal do Piauí, Teresina, PI, Brazil; Universidade de Pernambuco, Faculdade de Ciências Médicas, Recife, PE, Brazil; Instituto de Saúde Coletiva, Universidade Federal da Bahia, Salvador, BA, Brazil; Secretaria de Estado da Saúde de Alagoas, Maceió, AL, Brazil; Universidade Federal do Acre, Rio Branco, AC, Brazil; Universidade Federal do Maranhão, Departamento de Saúde Pública, São Luís, MA, Brazil; Universidade Federal de Sergipe, Aracaju, SE, Brazil; Secretaria Municipal de Saúde, Boa Vista, RR, Brazil; Fundação Oswaldo Cruz, Mato Grosso do Sul, Campo Grande, MS, Brazil; Fundação Oswaldo Cruz, Escola Nacional de Saúde Pública Sergio Arouca, Rio de Janeiro, RJ, Brazil; Universidade Federal do Rio Grande do Sul, Porto Alegre, RS, Brazil; Fundação Oswaldo Cruz, Instituto de Pesquisa René Rachou, Belo Horizonte, MG, Brazil; Secretaria de Desenvolvimento Ambiental de Rondônia, Porto Velho, RO, Brazil; Universidade de Brasília, Brasília, DF, Brazil

**Keywords:** Vacilación a la Vacunación, Programas de Inmunización, Factores Socioeconómicos, Cobertura de Vacunación, Encuestas Epidemiológicas, Vaccination Hesitancy, Immunization Programs, Socioeconomic Factors, Vaccination Coverage, Health Surveys

## Abstract

**Objective:**

To analyze vaccine hesitancy associated factors and repercussions on vaccination coverage.

**Methods:**

Cohort of children born in 2017-2018, living in Brazilian state capitals, Federal District (FD), and 12 inner region cities, stratified by socioeconomic level. National Vaccination Coverage Survey (2020) data on hesitancy, access and programmatic difficulties were obtained by interview and coverage was calculated from vaccination card dose and date records.

**Results:**

37801 children were studied, 31001 in the capitals/FD, 6800 in the inner cities. Hesitation between 38.8(95%CI 33.6;44.4) and 57.9(95%CI 54.1;61.6) in high versus low stratum; 64.1(95%CI 58.9;68.9) to 41.7(95%CI 38.4;45.8) among mothers with <8 years of schooling versus 16 years or more; 42.1(95%CI 38.2;46.2) to 55.0(95%CI 52.0;54.7) among private service users versus public service only users. Coverage: full=7.2(95%CI 1.0;38.3); 25.3(95%CI 18.7;33.3) for hesitant people; and 44.7(95%CI 43.0;46.4) for the remainder.

**Conclusion:**

High vaccine hesitancy in several groups affecting vaccination coverage and hindering vaccination target achievement. Access problems and programmatic difficulties contribute to low coverage.

## Study contributions

**Main results:**

Vaccine hesitancy has increased in Brazil, negatively impacting vaccination coverage. Socioeconomic strata of areas of residence, maternal schooling and family income are associated with hesitancy.

**Implications for services:**

Health service managers and workers need to know and understand the components of vaccine hesitancy, access problems and program difficulties in order to overcome low vaccination coverage among children.

**Perspectives:**

The problems are not specific to Brazil. Vaccine hesitancy is a growing phenomenon and needs appropriate management. Although vaccine refusal is not very common in Brazil, it may increase if hesitancy is not addressed.

## RESUMEN

**Objetivo:**

Analizar hesitación vacunal y repercusiones en la cobertura de vacunación.

**Métodos:**

Cohorte de niños nacidos en 2017-2018, residentes en capitales brasileñas, Distrito Federal y 12 ciudades del interior, estratificados por nivel socioeconómico. Datos de la Encuesta Nacional de Cobertura de Vacunación, 2020, sobre hesitación, acceso y dificultades programáticas obtenidos mediante entrevista y cobertura calculada con datos de la cartilla.

**Resultados:**

Incluidos 37.801 niños, 31.001 en las capitales/DF, 6.800 en el interior. Hesitación entre 38,8(IC95%:33,6-44,4) y 57,9(IC95%:54,1-61,6) estrato alto versus bajo; 64,1(IC95%:58,9-68,9) a 41,7(IC95%:38,4-45,8) madres <8 años de escolaridad versus ≥16 años; 42,1 (IC 95%: 38,2-46,2) y 55,0 (IC 95%: 52,0-54,7) utilizaron servicios privados versus solo servicios públicos. Cobertura completa 7,2(IC95%:1,0-38,3) y 25,3(IC95%:18,7-33,3) en hesitantes y 44,7(IC95%:43,0-46,4) en los demás.

**Conclusión:**

Alta hesitación vacunal en varios grupos afecta cobertura de vacunación, contribuyendo al fracaso en el logro de metas. Problemas de acceso y dificultades programáticas contribuyen a baja cobertura.

## INTRODUCTION

“The earth is round, gasoline is inflammable and vaccines are safe. Everything else is dangerous lies.” With this statement, in 2016 virologist Roberto Burioni, from the University of Milan, ended his brief participation in a debate on Italian television, in which the other two participants were a disc jockey and an actress, who were against vaccines.^1^

Most people recognize the benefits of vaccines, individually and collectively, and are satisfied with the way they are provided by health programs. A significant minority, ranging worldwide between 10% and 30%, have doubts about vaccines.^2^ Between 5% and 10% of people have strong anti-vaccine beliefs.^3^ It is difficult to have a clear view of vaccine hesitancy among the child population because there is no direct relationship between parents’ doubts and vaccination coverage. Those who are hesitant may accept all recommended vaccines even if they have significant doubts.^3^

At the beginning of the 21^st^ century, reductions in vaccination coverage began to be noticed in countries where coverage had traditionally been high. This period is characterized by low incidence of vaccine-preventable diseases, increase in the number and diversity of recommended vaccines, resurgence of articulated and well-funded anti-vaccine movements and speading of doubts about the safety and effectiveness of vaccines via the internet and social networks.^4^

Mass vaccination is a modern public health success story. The vaccination policy has been based on three principles: solidarity (citizens share collective responsibility for preventing preventable diseases); risk perception (being capable of identifying challenges and selecting effective means to face them); and acceptance of the need for institutional mechanisms to implement recommendations.^5^

The dynamics of vaccine refusal and resistance are different. The principles cited above are challenged. Self-protection becomes privileged to the detriment of solidarity, technical scientific knowledge is placed in doubt or simply denied and the quest for mechanisms for exemption from obligations exceeds recommendations.^5^

In 2019, the World Health Organization (WHO) included vaccine hesitancy among the top ten global health threats.^6^ There is disagreement about the way in which the WHO defined vaccine hesitancy. The organization attributed a significant part of this phenomenon to the reduction of vaccination coverage, mixing aspects related to parental decision-making, aspects of accessibility and organization of the immunization programs themselves. 

The term hesitation, which means indecision and vacillation, a psychological state that can delay action or result in inaction, has been used with three problematic meanings: behavior; individuals confident in their anti-vaccine stance; and incomplete vaccination resulting from reasons associated with access to services or program operation shortcomings.^7^

There are different actions that may or may not be associated with hesitancy: people who have no doubts or concerns and get vaccinated correctly; people who, despite doubts, get vaccinated correctly; people who have concerns and delay or select vaccines; and people who have no doubts and refuse all vaccines.^8^

In Brazil, vaccination coverage began to fall at the beginning of the 21^st^ century, initially among children from the highest socioeconomic classes, living in large urban centers. This drew attention to vaccine acceptance issues becoming more prominent than access difficulties, as a reason for not achieving protection targets.^9-10^

The recent drops in vaccination coverage in Brazil and the WHO’s concern about this issue were the justifications for including this topic in the 2020 National Vaccination Coverage Survey and for carrying out this study.

The objectives of this article are: to analyze some of the factors associated with vaccine hesitancy among those responsible for children born in 2017 and 2018 and who live in in Brazilian state capital cities, the Federal District and 12 other cities; and to verify whether vaccine hesitancy has an impact on vaccination coverage and whether there are barriers to access and programmatic difficulties faced by parents.

## METHODS

### Study design 

A retrospective cohort of children born alive in 2017 and 2018 was analyzed with regard to the first 24 months of their lives in relation to vaccine doses and vaccination dates recorded on their vaccination cards.

### Background

The census tracts of the 39 cities included in the survey were stratified according to socioeconomic indicators into four strata. In each stratum,^11^ children born in 2017 and 2018 were sampled, proportional to live births, by systematic selection of census tracts in each stratum and inclusion of all children in the cohort. The survey was carried out in 2020 and 2021 so that all children studied were at least 24 months old. 

Participants

The sample size was calculated at 40,228 children from four socioeconomic strata. Following losses (6%), 37,801 participants were included. For further details, see the published article on the survey methodology.^11^

### Variables associated with vaccine hesitancy

The variables associated with vaccine hesitancy were: socioeconomic strata of the census tracts of residence classified as high, medium, medium low and low; maternal education classified as up to 8 years of schooling, 9 to 12 years, 13 to 15 years and 16 years and more; and use of private services for administration of one or more doses of vaccine or exclusive use of public services to this end. 

### Vaccine hesitancy

Vaccine hesitancy was assessed regarding the parents’ intention to vaccinate (Has your child ever stopped being vaccinated due to your decision?) and their perception about vaccines by means of five questions, on a Likert scale,^12^ referring to the importance of vaccines for the child and the community, the need to vaccinate, trust and safety. Parents who expressed an intention not to vaccinate or to select only some vaccines, were asked to indicate reasons that influenced this intention. The hesitation questionnaire was proposed by the WHO, and responses were categorized dichotomously. Anyone who answered yes to at least one of the questions was considered hesitant.

### Vaccination coverage

Photographs of vaccination records were taken in order to check compliance with the planned schedule, according to valid dose criteria (given at the correct time and with the correct interval)^11^ for each child. Full coverage included all doses and vaccines provided for on the national vaccination schedule for each child.

Access to the program was investigated based on the reports of barriers to taking children for vaccination and specification of difficulties, including lack of documents, lack of time, vaccination service opening hours, distance, means of transport, financial resources, lack of knowledge of the schedule, physical disability or health problems.

Program operation difficulties were investigated by asking the question “Has your child ever failed to be vaccinated despite being taken to the vaccination center?” and specified according to lack of supplies, health professionals, scheduling, queues, lack of documentation and health professional recommendation not to administer several vaccines on the same day.

Other socioeconomic and demographic variables of the family or mothers were used for adjustment: *Bolsa Família* income transfer program beneficiary (yes, no), family income (up to BRL 1000; BRL 1001-3000; BRL 3001-8000; >BRL 8000), maternal age (<20 years, 20-34 years, 35 years and over), maternal employment (yes, no), maternal race/skin color (categories used by the Brazilian Institute of Geography and Statistics).

### Data source

Household interviews and photographs of vaccination cards were used as the data source.

### Statistical analysis 

Vaccine hesitancy for associated factors and odds ratios (OR) and respective 95% confidence intervals were calculated according to the proportion of hesitant people in each subgroup, with data obtained at the time of the interview.

Vaccination coverage at 24 months with administered doses and valid doses was calculated for hesitancy, accessibility and programmatic difficulties with respective 95% confidence intervals. Coverage levels were treated as incidence rates obtained longitudinally over the 24 month follow-up period. Relative risk (RR) and fractions attributable for exposure were calculated.

The relative risks and fractions attributable to exposure were calculated for the incomplete coverage rate at 24 months according to intention to vaccinate, perception about vaccines, access difficulties and program operation problems. Only statistically significant reasons are shown in the results, given the article length restrictions. 

All the analyses were performed using Stata 17.0, using the survey module for complex samples, weights calibrated for the population and sample losses. Poisson regression was used to adjust other socioeconomic variables in order to analyze the relationship between hesitation, access and programmatic difficulties across strata, maternal education and type of health service. The analysis was carried out by comparing the empty model and the complete model, with progressive removal of each variable and checking the fit, step by step. There was no selection of variables based on statistical significance in the bivariate analysis.

The survey was approved by the Research Ethics Committee of the *Instituto de Saúde Coletiva da Universidade Federal da Bahia*, as per Opinion No. 3.366.818, on June 4, 2019, and Certificate of Submission for Ethical Appraisal (*Certificado de Apresentação de Apreciação* Ética – CAAE) No. 4306919.5.0000.5030; and by the Research Ethics Committee of the *Irmandade da Santa Casa de São Paulo*, as per Opinion No. 4.380.019, on November 4, 2020, and CAAE No. 39412020.0.0000.5479.

## RESULTS 

A total of 37,801 children were included from the original sample of 40,228, distributed over the high (8,333), medium (9,418), low medium (9,992) and low (10,058) strata. There were 3,288 mothers with up to 8 years of schooling, 5,494 with 9-12 years, 15,623 with 13-15 years and 13,396 with 16 years or more of schooling. 29,265 children used the public vaccination service exclusively, while 8,536 used a private service for at least one vaccine. 19,777 people were hesitant, and 2,811 reported programmatic difficulties and access problems. Full coverage was found for 16,708 children.

Table 1 shows the proportion of vaccine hesitancy and ORs for each of the socioeconomic strata, maternal education and use of private services for vaccination. The data show that the proportion of parents and guardians who expressed vaccine hesitancy, whether in their intention to vaccinate or in their perceptions about vaccines, is significantly lower among those residing in the high and medium socioeconomic strata and higher for those residing in the lower strata. The values were high, ranging from 38.8% in the high stratum to 57.9% in the low stratum.

**Table 1:** Vaccine hesitancy of parents and guardians (per 100 children), with 95% confidence intervals (95%CI) and odds ratios (OR), according to social stratum, maternal schooling and use of private services, National Vaccination Coverage Survey, Brazil, 2020

Socioeconomic stratum	Number of children	Hesitation (per 100 children) and % (95%CI)	OR (95%CI)
High	8,333	38.8 (33.6;44.36)	0.87 (0.83;0.92)
Medium	94,118	46.9 (42.8;51.1)	0.89 (0.85;0.94)
Medium low	9,992	48.3 (45.21;51.5)	0.96 (0.92;1.00)
Low	10,058	57.9 (54.1;61.7)	1.28 (1.22;1.33)
Total	37,801	52.3 (49.9;54.7)	1.00
**Maternal schooling (years)**			
Up to 8	3,288	64.1 (58.9;68.9)	1.59 (1.47;1.71)
9-12	5,494	63.6 (58.9;68.0)	1.34 (1.26;1.42)
1-15	15,623	53.4 (50.3;56.4)	1.07 (1.03;1.12)
≥16	13,396	41.7 (38.4;45.8)	0.72 (0.69;0.75)
Total	37,801	52.1 (49.7;54.5)	1.00
**Use of private services**			
Yes	8,536	42.1 (38.2;46.1)	0.87 (0.83;0.91)
No	29,265	55.0 (52.2;57.7)	1.67 (1.61;1.72)
Total	37,801	52.3 (49.9;54.7)	1.00

There was an inversely proportional gradient with a higher proportion of hesitation among less educated mothers and a lower proportion among mothers with higher education levels. Using private vaccination services was associated with lower hesitancy. Exclusive use of public services was associated with greater hesitancy, although the proportions are high in both groups, 42.1% and 55.0%, respectively.

Poisson regression showed that socioeconomic strata and maternal education remain significant after adjustment for other sociodemographic variables, with relative risks of 1.06 (95%CI 1.01;1.12) and 0.91 (95%CI 0.87;0.95), respctively, while use of private services lost significance. The other economic variable that remained significant in the model was family income, with a relative risk of 0.92 (95%CI 0.88;0.96).

Table 2 compares coverage for the full scheme at 24 months, administered doses and valid doses, according to hesitation, access and programmatic difficulties. There was higher coverage for children in families that decided they should receive all vaccines, but even in this case, coverage was below National Immunization Program targets. Coverage was higher among those who did not show hesitation (intention and perception) and lower for families who reported access problems and programmatic difficulties. 

**Table 2:** Full vaccination coverage at 24 months (%) and 95% confidence intervals (95%CI) for administered and valid doses, according to vaccine hesistancy, difficulties in accessing services and program operation difficulties, National Vaccination Coverage Survey, Brazil, 2020

Exposure	Administered doses % (95%CI)	Valid doses % (95%CI)
Decision not to vaccinate	21.4 (4.2;62.7)	7.2 (1.0;38.3)
Decision to not give some vaccines	37.6 (29.5;46.4)	25.3 (18.7;33.4)
Decision to give all vaccines	60.7 (59.2;62.2)	44.7 (43.0;46.4)
Hesitant (intention and perception)	57.9 (55.8;60.1)	44.6 (42.3;46.9)
Non-hesitant (intention and perception)	62.4 (60.3;64.5)	43.7 (41.6;45.9)
Difficulty in accessing services	48.3 (43.4;53.2)	35.2 (30.4;40.3)
No difficulty in accessing services	61.0 (59.5;62.6)	44.8 (43.0;46.5)
Program operation difficulties	56.6 (53.9;59.2)	44.2 (41.6;46.8)
No program operation difficulties	61.2 (59.4;62.9)	44.1 (42.2;46.0)
Total	57.9 (55.8;60.1)	44.2 (42.5;45.9)

Regarding the full schedule with valid doses, all coverage levels were below 50%, being similar for those who decided to administer all vaccines, those who were hesitant and non-hesitant and those who faced programmatic difficulties. The lowest coverage was found for those who reported access problems, and those who decided to administer only some vaccines or no vaccine at all.

Table 3 shows incomplete coverage at 24 months, according to the components of vaccine hesitancy, showing the relative risks and the fractions attributable to exposure. The risk of having incomplete coverage was 32% higher than among those who decided to receive all vaccines, accounting for the 24% reduction in coverage. The most important reasons for the decision were the belief that vaccines are harmful to children’s health and fear of injections. The following stood out regarding perception about vaccines: believing that vaccines are not important for the child’s own health, believing that vaccines do not contribute to the health of children in the neighborhood and not trusting vaccines distributed by the government. 

**Table 3:** Incomplete vaccination coverage (%), relative risk (RR) and attributable fraction, with 95% confidence intervals (95%CI), among those exposed (%), according to vaccine hesistancy components, National Vaccination Coverage Survey, Brazil, 2020

Hesitation	Incomplete coverage % (95%CI)	RR (95%CI)	Attributable fraction among the exposed % (95%CI)
**Intention and reasons**			
I do not give any vaccines or only give some of them	74.4 (29.1;80.1)	1.32 (1.22;1.42)	24.10 (18.16;29.61)
I believe that vaccines are bad for health	85.8 (69.9;94.0)	3.66 (3.01;4.44)	0.72 (0.66;0.77)
I am afraid of injections	88.0 (74.4;94.9)	1.21 (1.12;1.30)	17.71 (11.33;23.64)
**Perception**			
Vaccines are not important for my child’s health	61.0 (52.8;68.6)	1.08 (1.04;1.13)	8.06 (3.85;12.09)
Vaccines do not contribute to the health of the children in the neighborhood	56.7 (49.8;63.4)	1.21 (1.17;1.26)	17.85 (14.91;20.68)
I do not trust the vaccines distributed by the government	56.8 (52.9;60.7)	1.06 (1.04;1.09)	6.21 (3.91;8.45)

Table 4 shows incomplete coverage, relative risks and attributable fractions for the main access difficulties and programmatic problems. The two most outstanding reasons that influenced access were lack of money for transportation to the vaccination service and the loss of the child’s vaccination card. These reasons, associated with incomplete coverage, explain 62% of the reduction in coverage. Lack of time and not knowing when to take the child for vaccination were also significant. 

**Table 4:** Incomplete vaccination coverage at 24 months (%), relative risk (RR) and attributable fraction among those exposed (%) with 95% confidence intervals (95%CI), according to difficultiesin accessing vaccination services or program operation difficulties, National Vaccination Coverage Survey, Brazil, 2020

Reasons	Incomplete coverage % (95%CI)	RR (95%CI)	Attributable fraction among the exposed % (95%CI)
Dificulty in access	64.7 (59.7;69.6)	1.14 (1.10;1.17)	12.35 (9.70;14.92)
Lost the vaccination card	91.4 (81.7;96.3)	1.26 (1.15;1.39)	21.03 (13.13;28.20)
Lack of time to take child to health center	61.6 (53.0;69.7)	1.09 (1.03;1.16)	8.79 (3.57;14.08)
Has no money for transport	69.3 (56.2;80.0)	1.69 (1.56;1.82)	40.91 (36.22;45.25)
Does not know when to take child to health center	67.0 (54.2;77.7)	1.19 (1.09;1.30)	16.33 (8.94;23.12)
Went to health center, but was unable to vaccinate	55.8 (53.2;58.4)	0.99 (0.97;1.01)	0.39 (0.00;2.35)
Long waiting time	53.9 (46.7;61.0)	1.07 (1.01;1.12)	6.56 (1.88;11.01)
Did not have all the documents required by health center	59.3 (49.9;68.2)	1.15 (1.09;1.23)	13.75 (8.32;18.86)
Health professional recommended not having several different vaccinations on the same day	60.4 (54.5;66.2)	1.07 (1.02;1.12)	6.75 (2.33;10.97)

In the case of reasons associated with missed opportunities, the most relevant reason was lack of documents, accounting for a 13% reduction in coverage. Also noteworthy is vaccination room health professionals not recommending simultaneous administration of vaccines, as well as problems with queues.

## DISCUSSION

Vaccine hesitancy is a complex phenomenon that encompasses parent’s perceptions and intentions about. In the survey conducted in 39 Brazilian cities, which included all regions of Brazil, high proportions of vaccine hesitancy were found in all social classes. Hesitation is more apparent in lower socioeconomic strata, in families with lower education and income, as found in Canada.^13^

The data showed the importance of access issues, exceeding hesitancy as a reason for lower vaccination coverage. Programmatic issues appeared as reasons for missed opportunities, although apparently with less impact on vaccination coverage. 

A science and health perception survey conducted in 140 countries in 2016 showed that 79% of people agree that vaccines are safe, with wide variation between regions of the world. In Latin America, 63% agree that vaccines are safe, 82% that they are effective and 97% believe that vaccines are important for health.^14^

A survey carried out in 67 countries in 2016 to assess trust regarding vaccines found great variability in answers to questions relating to importance, safety and effectiveness. Men gave less importance to vaccines, while adults and the elderly recognized their effectiveness more. There were no differences regarding education and income, but negative feelings about safety were more common in high-income countries, with higher gross domestic product per capita, higher spending on health and higher levels of education.^15^

Empirical studies on hesitation have shown that the phenomenon is context specific, with no predictors that can be seen in all situations.^16^ The data from our study show less hesitation in higher social strata, with higher education and higher income, differing from previous studies carried out in Brazil and in cities like São Paulo.^17,18^

Differences found between the middle class and the working class in the United States in 2018^19^ show that refusal of all or some vaccines is more frequent among middle-class, White families, with annual income above US$70,000 and with higher education. Working-class families, with non-Anglo-Saxon parents, without higher education and below the poverty line, have more children with an incomplete vaccination schedule due to reasons associated with access barriers, cost, logistical issues and lower health insurance coverage. 

There is a wide spectrum of attitudes about vaccines, including those who are pro-vaccine and accept all of them, those who have many doubts but fully or partially vaccinate their children, and those few who refuse vaccines altogether.^20^ Our study corroborates these findings by showing that, even among those who stated that they did not intend to vaccinate their children, there are children with a full vaccination schedule. Vaccine hesitancy, expressed by the decision to vaccinate completely or partially, or not to vaccinate, hinders achievement of desired coverage levels. Access barriers and program difficulties form a complex picture of circumstances that contribute to drops in coverage in practically all countries.

Different conceptual models have been proposed with the aim of understanding the components of the parental decision-making process.Dubé et al. (2013) proposed a broad set of determinants grouped into: historical, political and sociocultural context, vaccination policies, communication and media and health professionals.^3^ In 2018, these authors formulated an ecological-social model rearranging the determinants according to hierarchical levels: vaccination policy (supply, costs, schedule), community (social norms, sociocultural perspectives on health and vaccines), organizational level (services, providers and health professionals), interpersonal level (family, friends, social networks and lifestyles) and individual level (knowledge, beliefs, attitudes, values and sociodemographic characteristics).^16,21^

Social factors such as knowledge, past experiences, perception of the importance of vaccines, perception of risk and trust, subjective norms and religious convictions influence the decision to vaccinate.^3,22^ Seeing vaccination as a social norm is an important determinant of acceptance as well as social responsibility.^22^ The postmodern context of questioning the validity of science, competence and medical and health authorities favors questioning and skepticism.^16^ Our study indicates that people who do not believe that vaccinating their child is important for ensuring the health of other children in the neighborhood are more likely to have incomplete schedules.

Contradictorily, the focus of health promotion centered on healthy lifestyles, contributes to doubts about the importance and need for vaccines, as well as adherence to different complementary or alternative practices.^3,9,23,24^ Many parents believe that it is preferable for children to contract diseases, as this would give them long-lasting protection without overloading their immune system. Although they tend to consider the importance of vaccination from a more general perspective, they do not believe that it is necessary for their children, based on the assumption that they would be protected by a healthy lifestyle.^9,24,25^

Dimensions of risk perception, such as vulnerability and severity of consequences if harm occurs, can bias towards vaccination and omission, leading to delay, choice of vaccines or even refusal. As the risks represented by adverse effects are immediately detectable in individuals and the benefits are more difficult to note individually, perception of risk may lean towards hesitation rather than acceptance. Use of data from adverse effect reporting systems without due care to verify proof of the causal link is often used to fuel fake news and concern about the safety of vaccines.^2,3,^ ^24,25,26,27,28^

Lack of knowledge about who, where and when to vaccinate also appears among the reasons for not having the full schedule, both in this study and in others. Accessibility and convenience favor acceptance, while negative experiences with services increase rejection.^3,25^ The increase in the number of vaccines and the different regimens used can increase negative perception about the need and relevance of vaccines.^3,^ ^24,25^

Healthcare professionals are the main sources of vaccine guidance for parents. Positive attitudes towards vaccines influence acceptance by parents.^29,30^ It is very important that they are adequately prepared to discuss doubts and provide safe guidance. Many professionals have little knowledge about vaccines, timing, importance and veracity of adverse effects.^3,27^ This study indicates that guidance given by vaccination room professionals that is not in keeping with the schedule proposed, avoiding the simultaneous administration of several vaccines, is associated with greater likelihood of incomplete schedules. This increases the number of visits to the vaccination service needed to keep to the schedule and can lead to invalid doses when disregarding the timing of vaccination and the interval between doses. 

The limitations of this study are related to the inherent difficulty of quantitative procedures when trying to understand complex social phenomena. It is possible to quantify the stratifications, but the apparent contradictions are often not understood. 

The intention not to vaccinate was associated with incomplete coverage at 24 months, as well as lack of trust and the perception that vaccines are bad for health and are not important either for the child in question or for other children in the neighborhood. Access problems are more important as reasons for low coverage than issues of vaccine hesitancy, highlighting lack of time and money, loss of vaccination cards and lack of knowledge about when to take the child for vaccination. Program performance problems also end up contributing to lower coverage, notably queues, lack of documents and recommendations made by vaccination room health workers not to simultaneously administer all doses as per the vaccination schedule. 
