# Supplementary material for: Vaccine hesitancy and consequences for vaccination coverage in children at 24 months of age, born in 2017-2018, living in the state capitals, Federal District and 12 inner region cities of Brazil
Source: Epidemiol Serv Saude. 2025 Jan 10;33(spe2):e20231097. doi: 10.1590/S2237-96222024v33e20231097.especial2.en (PMC11734594; doi:10.1590/S2237-96222024v33e20231097.especial2.en)
Supplement: Translation

# Translation: Hesitação vacinal e consequências para a cobertura vacinal em crianças aos 24 meses de idade, 2017-2018, residentes nas capitais, no Distrito Federal e em 12 cidades do interior do Brasil

**DOI:** 10.1590/S2237-96222024v33e20231097.especial2.pt

**Authors:** Rita Barradas Barata, Ana Paula França, Ione Aquemi Guibu, Gabriel Munhoz, Carla Magda Allan Santos Domingues, Maria da Gloria Teixeira, José Cássio de Moraes, Grupo ICV 2020, Adriana Ilha da Silva, Alberto Novaes Ramos, Ana Paula França, Andrea de Nazaré Marvão Oliveira, Antonio Fernando Boing, Carla Magda Allan Santos Domingues, Consuelo Silva de Oliveira, Ethel Leonor Noia Maciel, Ione Aquemi Guibu, Isabelle Ribeiro Barbosa Mirabal, Jaqueline Caracas Barbosa, Jaqueline Costa Lima, José Cássio de Moraes, Karin Regina Luhm, Karlla Antonieta Amorim Caetano, Luisa Helena de Oliveira Lima, Maria Bernadete de Cerqueira Antunes, Maria da Gloria Teixeira, Maria Denise de Castro Teixeira, Maria Fernanda de Sousa Oliveira Borges, Rejane Christine de Sousa Queiroz, Ricardo Queiroz Gurgel, Rita Barradas Barata, Roberta Nogueira Calandrini de Azevedo, Sandra Maria do Valle Leone de Oliveira, Sheila Araújo Teles, Silvana Granado Nogueira da Gama, Sotero Serrate Mengue, Taynãna César Simões, Valdir Nascimento, Wildo Navegantes de Araújo

**Affiliations:** 1 Faculdade de Ciências Médicas da Santa Casa de São Paulo, Departamento de Saúde Coletiva, São Paulo, SP, Brasil; 2 Organização Pan-Americana da Saúde, Brasília, DF, Brasil; 3 Universidade Federal da Bahia, Instituto de Saúde Coletiva, Salvador, BA, Brasil; Universidade Federal do Espírito Santo, Vitória, ES, Brasil; Universidade Federal do Ceará, Departamento de Saúde Comunitária, Fortaleza, CE, Brasil; Faculdade Ciências Médicas Santa Casa de São Paulo, São Paulo, SP, Brasil; Secretaria de Estado da Saúde do Amapá, Macapá, AP, Brasil; Universidade Federal de Santa Catarina, SC, Brasil; Organização Pan-Americana da Saúde, Brasília, DF, Brasil; Instituto Evandro Chagas, Belém, PA, Brasil; Faculdade de Ciências Médicas Santa Casa de São Paulo, Departamento de Saúde Coletiva, São Paulo, SP, Brasil; Universidade Federal de Mato Grosso, Cuiabá, MT, Brasil; Universidade Federal do Paraná, Curitiba, PR, Brasil; Universidade Federal de Goiás, Goiânia, GO, Brasil; Universidade Federal do Piauí, Teresina, PI, Brasil; Universidade de Pernambuco, Faculdade de Ciências Médicas, Recife, PE, Brasil; Instituto de Saúde Coletiva, Universidade Federal da Bahia, Salvador, BA, Brasil; Secretaria de Estado da Saúde de Alagoas, Maceió, AL, Brasil; Universidade Federal do Acre, Rio Branco, AC, Brasil; Universidade Federal do Maranhão, Departamento de Saúde Pública, São Luís, MA, Brasil; Universidade Federal de Sergipe, Aracaju, SE, Brasil; Secretaria Municipal de Saúde, Boa Vista, RR, Brasil; Fundação Oswaldo Cruz, Mato Grosso do Sul, Campo Grande, MS, Brasil; Fundação Oswaldo Cruz, Escola Nacional de Saúde Pública Sergio Arouca, Rio de Janeiro, RJ, Brasil; Universidade Federal do Rio Grande do Sul, Porto Alegre, RS, Brasil; Fundação Oswaldo Cruz, Instituto de Pesquisa René Rachou, Belo Horizonte, MG, Brasil; Secretaria de Desenvolvimento Ambiental de Rondônia, Porto Velho, RO, Brasil; Universidade de Brasília, Brasília, DF, Brasil

**Keywords:** Hesitação Vacinal, Acesso aos Serviços de Saúde, Programa de Imunização, Fatores Socioeconômicos, Cobertura Vacinal

## Abstract

**Objetivo:**

Analisar fatores associados à hesitação vacinal e repercussões nas coberturas vacinais.

**Métodos:**

Utilizou-se coorte de crianças nascidas em 2017 e 2018, residentes nas capitais brasileiras, no Distrito Federal e em 12 cidades do interior, estratificadas por nível socioeconômico. Dados do Inquérito Nacional de Cobertura Vacinal de 2020 sobre hesitação, acesso e dificuldades programáticas foram obtidos por entrevistas e coberturas, calculadas com doses e datas da caderneta.

**Resultados:**

Foram estudadas 37.801 crianças, sendo 31.001 nas capitais e no Distrito Federal e 6.800 no interior. Hesitação entre 38,8 (IC_95_% 33,6;44,4) e 57,9 (IC_95_% 54,1;61,6) estrato alto *versus* baixo; 64,1 (IC_95_% 58,9;68,9) a 41,7 (IC_95_% 38,4;45,8) mães com até 8 anos de escolaridade *versus* 16 anos ou mais; 42,1 (IC_95_% 38,2;46,2) e 55,0 (IC_95_%52,0;54,7) usaram serviços privados *versus* usaram serviços públicos. Cobertura completa 7,2 (IC_95_% 1,0;38,3) e 25,3 (IC_95_% 18,7;33,3) nos hesitantes e 44,7 (IC_95_% 43,0;46,4) nos demais.

**Conclusão:**

A hesitação vacinal alta em vários grupos afeta as coberturas vacinais e prejudica o alcance das metas de vacinação. Problemas de acesso e dificuldades programáticas concorrem para baixas coberturas.

## Contribuições do estudo

**Principais resultados:**

A hesitação vacinal aumentou no Brasil, impactando negativamente as coberturas vacinais. Estratos socioeconômicos das áreas de residência, escolaridade materna e renda familiar são associados à hesitação.

**Implicações para os serviços:**

Os gestores e profissionais precisam conhecer e entender os componentes da hesitação vacinal, os problemas de acesso e as dificuldades do programa para superar as baixas coberturas vacinais em crianças.

**Perspectivas:**

Os problemas não são específicos do Brasil. A hesitação vacinal é um fenômeno em crescimento e precisa de manejo adequado. Embora a recusa a vacinas não não ser muito presente no Brasil, pode aumentar se a hesitação não for enfrentada.

## INTRODUÇÃO

“A terra é redonda, gasolina é inflamável e vacinas são seguras. Tudo o mais são mentiras perigosas”. Com essa afirmação, o virologista Roberto Burioni, da Universidade de Milão, em 2016, encerrou sua breve participação em um debate na televisão italiana, no qual os outros dois participantes eram um disc jockey e uma atriz, contrários às vacinas.^1^

A maioria das pessoas reconhece os benefícios das vacinas, individual e coletivamente, e estão satisfeitas com a forma como elas são oferecidas pelos programas de saúde. Uma minoria significativa, variando mundialmente entre 10% e 30%, tem dúvidas relativas às vacinas.^2^ Entre 5% e 10% das pessoas têm fortes convicções antivacina.^3^ É difícil ter uma visão clara sobre a hesitação vacinal, no âmbito populacional, porque não há relação direta entre as dúvidas dos pais e as coberturas vacinais. Os hesitantes podem aceitar todas as vacinas recomendadas ainda que tenham dúvidas significativas.^3^

No início do século XXI, começaram a ser observadas reduções nas coberturas vacinais em países onde tradicionalmente as coberturas eram altas. Esse período é caracterizado pela baixa incidência das doenças imunopreveníveis, pelo aumento no número e na diversidade de vacinas recomendadas, pelo ressurgimento de movimentos antivacina articulados e bem financiados e pela difusão de dúvidas sobre segurança e efetividade das vacinas através da internet e das redes sociais.^4^

A vacinação em massa é uma história de sucesso da saúde pública moderna. A política de vacinação esteve baseada em três princípios: a solidariedade (os cidadãos compartilham a responsabilidade coletiva pela prevenção de doenças evitáveis); a percepção do risco (esta é capaz de identificar os desafios e selecionar os meios efetivos para enfrentá-los); e a aceitação da necessidade de mecanismos institucionais, para implementar as recomendações.^5^

Há uma dinâmica diferente na recusa e resistência às vacinas. Os princípios anteriores são desafiados. A autoproteção passa a ser privilegiada em detrimento da solidariedade, o conhecimento técnico científico é colocado em dúvida ou simplesmente negado e a busca por mecanismos de isenção das obrigatoriedades sobrepassa as recomendações.^5^

Em 2019, a Organização Mundial de Saúde (OMS) incluiu a hesitação vacinal entre as 10 principais ameaças globais à saúde.^6^ Existe discordância sobre a maneira como a OMS definiu hesitação vacinal. A organização atribuiu a este fenômeno parte significativa na redução das coberturas vacinais, mesclando aspectos relativos à tomada de decisão dos pais, aspectos de acessibilidade e organização dos próprios programas de imunização. 

O termo hesitação, que significa indecisão e vacilação, estado psicológico que pode retardar a ação ou resultar em inação, vem sendo usado em três acepções problemáticas: comportamento; indivíduos seguros de sua posição antivacina; e vacinação incompleta decorrente de motivos associados a acesso ou falhas na execução de programas.^7^

Há diferentes ações que podem estar associadas ou não à hesitação: pessoas que não têm dúvidas ou preocupações e se vacinam corretamente; pessoas que, apesar das dúvidas, se vacinam corretamente; pessoas que têm preocupações e atrasam ou selecionam vacinas; e pessoas que não têm dúvidas e recusam todas as vacinas.^8^

No Brasil, no início do século XXI, as coberturas vacinais começaram a cair, inicialmente entre crianças das classes socioeconômicas mais altas, residentes em grandes centros urbanos. Isso chamou atenção para a substituição das dificuldades de acesso pelas questões de aceitação das vacinas, como motivo para o não alcance das metas de proteção.^9-10^

As quedas recentes nas coberturas vacinais no país e a preocupação da OMS com o tema foram as justificativas para a inclusão desse tópico no Inquérito Nacional de Cobertura Vacinal de 2020 e para a realização deste estudo.

Os objetivos deste artigo são: analisar alguns dos fatores associados à hesitação vacinal entre responsáveis por crianças nascidas em 2017 e 2018, residentes nas capitais brasileiras, no Distrito Federal e em 12 municípios; e verificar se a hesitação vacinal tem repercussão sobre as coberturas vacinais e se há barreiras ao acesso e dificuldades programáticas enfrentadas pelos pais.

## MÉTODOS

### Desenho de estudo

Analisou-se coorte retrospectiva dos nascidos vivos em 2017 e 2018, acompanhados durante os primeiros 24 meses de vida por meio dos registros das doses e datas de vacinas na caderneta de vacinação.

### Contexto

Os setores censitários das 39 cidades incluídas foram estratificados segundo os indicadores socioeconômicos em quatro estratos. Em cada estrato, foram amostradas^11^ crianças nascidas em 2017 e 2018, proporcional aos nascidos vivos, por sorteio sistemático dos setores em cada estrato e inclusão de todas as crianças da coorte. O inquérito foi realizado em 2020 e 2021 para que todas as crianças estudadas tivessem, no mínimo, 24 meses de idade. 

### Participantes

O tamanho da amostra foi calculado em 40.228 crianças dos quatro estratos socioeconômicos. Foram incluídos 37.801 participantes em função das perdas (6%). Para mais detalhes, consultar o artigo metodológico já publicado.^11^

### Variáveis associadas à hesitação vacinal

As variáveis associadas à hesitação vacinal foram: estratos socioeconômicos dos setores de residência classificados em alto, médio, médio baixo e baixo; escolaridade materna classificada em até 8 anos de escolaridade, 9 a 12 anos, 13 a 15 anos e 16 anos e mais; e uso de serviços privados para a aplicação de uma ou mais doses de vacina ou uso exclusivo dos serviços públicos. 

### Hesitação vacinal

A hesitação vacinal foi avaliada quanto à intenção de vacinar dos pais (Alguma vez a sua criança deixou de ser vacinada por sua decisão?) e à percepção sobre vacinas por meio de cinco perguntas, em escala Likert,^12^ referentes à importância das vacinas para a criança e a comunidade, à necessidade de vacinar, à confiança e à segurança. Para os pais que expressaram intenção de não vacinar ou de selecionar algumas vacinas, perguntaram-se os motivos que influenciaram essa intenção. O questionário sobre hesitação foi o proposto pela OMS, e as respostas foram categorizadas dicotomicamente. Considerou-se hesitante quem respondeu sim a pelo menos uma das perguntas.

### Cobertura vacinal

Foram obtidas fotografias das cadernetas de vacinação permitindo estabelecer o cumprimento do calendário previsto, segundo critérios de doses válidas (na época correta e com intervalo correto)^11^ para cada criança. A cobertura completa incluiu todas as doses e vacinas previstas no calendário nacional de vacinação para cada criança.

O acesso ao programa foi investigado a partir do relato de barreiras para levar a criança para vacinar e da especificação das dificuldades incluindo falta de documentos, falta de tempo, horário de funcionamento das unidades, distância, meios de transporte, recursos financeiros, desconhecimento do esquema, deficiência física ou problemas de saúde.

As dificuldades de funcionamento do programa foram investigadas por meio da pergunta “Alguma vez sua criança deixou de ser vacinada apesar de ter sido levada ao posto de vacinação?” e especificadas segundo falta de insumos, profissionais, agendamento, filas, ausência de documento e recomendação profissional para não aplicar várias vacinas no mesmo dia.

Outras variáveis socioeconômicas e demográficas da família ou das mães foram usadas para ajuste: Bolsa Família (sim, não), renda familiar (até R$ 1.000,00; R$ 1.001,00-R$ 3.000,00; R$ 3.001,00-R$ 8.000,00; >R$ 8.000,00), idade materna (<20 anos, 20-34 anos, 35 anos e mais), trabalho materno (sim, não), raça/cor da pele da mãe (categorias do Instituto Brasileiro de Geografia e Estatística).

### Fonte dos dados

Entrevistas realizadas nos domicílios e fotografias das cadernetas de vacinação foram utilizadas como fonte de dados.

### Análise estatística

A hesitação vacinal para os fatores associados e as razões de chance (*odds ratio*, OR) e os respectivos intervalos de confiança de 95% foram calculados conforme a proporção de hesitantes em cada subgrupo, com dados do momento da entrevista.

As coberturas vacinais aos 24 meses com doses aplicadas e doses válidas foram calculadas para hesitação, acessibilidade e dificuldades programáticas com os respectivos intervalos de confiança de 95%. Foram tratadas as coberturas como incidências obtidas longitudinalmente ao longo dos 24 meses de seguimento. Calculou-se o risco relativo (RR) e as frações atribuíveis às exposições.

Os riscos relativos e as frações atribuíveis à exposição foram calculados para a taxa de cobertura incompleta aos 24 meses segundo a intenção de vacinar, a percepção sobre vacinas, as dificuldades de acesso e os problemas operacionais do programa. Apenas os motivos estatisticamente significantes são apresentados nos resultados, dadas as restrições de extensão do artigo. 

Todas as análises foram realizadas no programa Stata 17.0, com o módulo *survey* para amostras complexas, pesos calibrados para a população e perdas amostrais. A regressão de Poisson foi utilizada para ajustar outras variáveis socioeconômicas para análise da relação entre hesitação, acesso e dificuldades programáticas aos estratos, escolaridade materna e tipo de serviço de saúde. A análise foi realizada por meio da comparação entre o modelo vazio e o modelo completo, com retirada progressiva de cada variável e verificação do ajuste, passo a passo. Não houve seleção das variáveis baseada na significância estatística na análise bivariada.

O estudo foi aprovado pelos comitês de ética em pesquisa com seres humanos do Instituto de Saúde Coletiva da Universidade Federal da Bahia, sob Parecer nº 3.366.818, em 4 de junho de 2019, com Certificado de Apresentação de Apreciação Ética (CAAE) 4306919.5.0000.5030; e da Irmandade da Santa Casa de São Paulo, sob Parecer nº 4.380.019, em 4 de novembro de 2020, com CAAE 39412020.0.0000.5479.

## RESULTADOS 

Da amostra de 40.228 foram incluídas 37.801 crianças, distribuídas nos estratos alto (8.333), médio (9.418), médio baixo (9.992) e baixo (10.058). As mães com até 8 anos de escolaridade foram 3.288, 5.494 com 9 a 12 anos, 15.623 com 13 a 15 anos e 13.396 com 16 anos ou mais. Usaram exclusivamente o serviço público de vacinação 29.265 crianças, e 8.536 usaram o serviço privado pelo menos para uma vacina. Eram hesitantes 19.777 responsáveis, e 2.811 referiram dificuldades programáticas e problemas de acesso. A cobertura completa foi observada para 16.708 crianças.

A Tabela 1 apresenta a proporção de hesitação vacinal e as OR para cada um dos estratos socioeconômicos, escolaridade materna e utilização de serviços privados para vacinação. Os dados mostram que a proporção de responsáveis que expressou hesitação vacinal, seja na intenção de vacinar, seja nas percepções sobre vacina, é significantemente menor entre os que residem nos estratos socioeconômicos alto e médio e maior para os que residem no estrato baixo. Os valores são altos variando de 38,8% no estrato alto a 57,9% no estrato baixo.

**Tabela 1:** Hesitação vacinal dos responsáveis (por 100 crianças), com intervalo de confiança (IC95%) e razão de chances (OR), segundo estrato social, escolaridade materna e uso de serviços privados, Inquérito Nacional de Cobertura Vacinal, Brasil, 2020

Estrato socioeconômico	Número de crianças	Hesitação (por 100 crianças) e (IC_95_ **%**)	OR (IC_95_ **%**)
Alto	8.333	38,8 (33,6;44,36)	0,87 (0,83;0,92)
Médio	94.118	46,9 (42,8;51,1)	0,89 (0,85;0,94)
Médio baixo	9.992	48,3 (45,21;51,5)	0,96 (0,92;1,00)
Baixo	10.058	57,9 (54,1;61,7)	1,28 (1,22;1,33)
Total	37.801	52,3 (49,9;54,7)	1,00
**Escolaridade materna (anos)**			
Até 8	3.288	64,1 (58,9;68,9)	1,59 (1,47;1,71)
9-12	5.494	63,6 (58,9;68,0)	1,34 (1,26;1,42)
1-15	15.623	53,4 (50,3;56,4)	1,07 (1,03;1,12)
≥16	13.396	41,7 (38,4;45,8)	0,72 (0,69;0,75)
Total	37.801	52,1 (49,7;54,5)	1,00
**Uso de serviços privados**			
Sim	8.536	42,1 (38,2;46,1)	0,87 (0,83;0,91)
Não	29.265	55,0 (52,2;57,7)	1,67 (1,61;1,72)
Total	37.801	52,3 (49,9;54,7)	1,00

Há gradiente inversamente proporcional com proporção maior de hesitação entre as mães menos escolarizadas e menor entre as mães com educação superior. O uso de serviços privados para vacinar está associado à menor hesitação. O uso exclusivo de serviços públicos está associado à maior hesitação, ainda que as proporções sejam altas nos dois grupos, 42,1% e 55,0%.

A regressão de Poisson mostrou que os estratos socioeconômicos e a escolaridade materna permanecem significantes após o ajuste por outras variáveis sociodemográficas, com riscos relativos de 1,06 (IC_95_% 1,01;1,12) e 0,91 (IC_95_% 0,87;0,95), enquanto o uso de serviços privados perde significância. A outra variável econômica que permanece significante no modelo é a renda familiar com risco relativo de 0,92 (IC_95_% 0,88;0,96).

A Tabela 2 compara as coberturas pelo esquema completo aos 24 meses, doses aplicadas e doses válidas, segundo hesitação, acesso e dificuldades programáticas. Há coberturas mais altas para as crianças das famílias que decidiram dar todas as vacinas, mas, mesmo nessas, a cobertura está abaixo dos objetivos do Programa Nacional de Imunizações. As coberturas são mais altas entre os que não apresentaram hesitação (intenção e percepção) e menores para as famílias que relataram problemas de acesso e dificuldades programáticas. 

**Tabela 2:** Cobertura vacinal completa aos 24 meses (%) e intervalo de confiança de 95% (IC95%) de doses aplicadas e válidas, segundo hesitação vacinal, dificuldades de acesso aos serviços e dificuldades de funcionamento do programa, Inquérito Nacional de Cobertura Vacinal, Brasil, 2020

Exposição	Doses aplicadas (IC_95_ **%**)	Doses válidas (IC_95_ **%**)
Decisão de não vacinar	21,4 (4,2;62,7)	7,2 (1,0;38,3)
Decisão de não dar algumas vacinas	37,6 (29,5;46,4)	25,3 (18,7;33,4)
Decisão de dar todas as vacinas	60,7 (59,2;62,2)	44,7 (43,0;46,4)
Hesitantes (intensão e percepção)	57,9 (55,8;60,1)	44,6 (42,3;46,9)
Não hesitantes (intensão e percepção)	62,4 (60,3;64,5)	43,7 (41,6;45,9)
Dificuldade de acesso a serviços	48,3 (43,4;53,2)	35,2 (30,4;40,3)
Sem dificuldade de acesso a serviços	61,0 (59,5;62,6)	44,8 (43,0;46,5)
Dificuldade de funcionamento do programa	56,6 (53,9;59,2)	44,2 (41,6;46,8)
Sem dificuldade de funcionamento do programa	61,2 (59,4;62,9)	44,1 (42,2;46,0)
Total	57,9 (55,8;60,1)	44,2 (42,5;45,9)

Para o esquema completo com doses válidas, todas as coberturas foram inferiores a 50%, sendo semelhantes para os que decidiram dar todas as vacinas, hesitantes e não hesitantes e com dificuldades programáticas. As menores coberturas foram observadas para os que referiram problemas de acesso, decisão de aplicar apenas algumas vacinas ou nenhuma vacina.

A Tabela 3 apresenta a cobertura incompleta aos 24 meses, de acordo com as dimensões da hesitação vacinal, mostrando os riscos relativos e as frações atribuíveis à exposição. O risco de ter cobertura incompleta foi 32% maior do que entre aqueles que decidiram dar todas as vacinas, respondendo pela redução de 24% na cobertura. Os motivos mais importantes na decisão foram a crença de que as vacinas fazem mal à saúde da criança e o medo de injeções. Na percepção sobre vacinas, o que se destacou foi: acreditar que as vacinas não são importantes para a saúde da própria criança, entender que as vacinas não contribuem para a saúde das crianças do bairro e não confiar nas vacinas distribuídas pelo governo. 

**Tabela 3:** Cobertura vacinal incompleta (%), risco relativo (RR) e fração atribuível, com intervalo de confiança (IC95%), entre os expostos (%), segundo componentes da hesitação vacinal, Inquérito Nacional de Cobertura Vacinal, Brasil, 2020

Hesitação	Cobertura incompleta % (IC_95_ **%**)	RR (IC_95_ **%**)	Fração atribuível entre os expostos % (IC_95_ **%**)
**Intenção e motivos**			
Não dou nenhuma vacina ou dou apenas algumas	74,4 (29,1;80,1)	1,32 (1,22;1,42)	24,10 (18,16;29,61)
Acredito que vacinas fazem mal à saúde	85,8 (69,9;94,0)	3,66 (3,01;4,44)	0,72 (0,66;0,77)
Tenho medo das injeções	88,0 (74,4;94,9)	1,21 (1,12;1,30)	17,71 (11,33;23,64)
**Percepção**			
Vacinas não são importantes para a saúde da criança	61,0 (52,8;68,6)	1,08 (1,04;1,13)	8,06 (3,85;12,09)
Vacinas não contribuem para a saúde das crianças do bairro	56,7 (49,8;63,4)	1,21 (1,17;1,26)	17,85 (14,91;20,68)
Não confio nas vacinas distribuídas pelo governo	56,8 (52,9;60,7)	1,06 (1,04;1,09)	6,21 (3,91;8,45)

A Tabela 4 apresenta a cobertura incompleta, os riscos relativos e as frações atribuíveis para as principais dificuldades de acesso e problemas programáticos. Os dois motivos mais evidentes que influenciaram o acesso foram a falta de dinheiro para o transporte até a unidade básica e a perda da caderneta. Esses motivos, associados à cobertura incompleta, explicam 62% da redução na cobertura. Foram significantes também a falta de tempo e não saber quando levar a criança para vacinar. 

**Tabela 4:** Cobertura vacinal incompleta aos 24 meses (%), risco relativo (RR) e fração atribuível entre os expostos (%) com intervalo de confiança (IC95%), segundo dificuldades de acesso aos serviços de vacinação ou dificuldades com o funcionamento do programa, Inquérito Nacional de Cobertura Vacinal, Brasil, 2020

Motivos	Cobertura incompleta % (IC_95_ **%**)	RR (IC_95_ **%**)	Fração atribuível entre os expostos % (IC_95_ **%**)
Dificuldade de acesso	64,7 (59,7;69,6)	1,14 (1,10;1,17)	12,35 (9,70;14,92)
Perdeu a caderneta	91,4 (81,7;96,3)	1,26 (1,15;1,39)	21,03 (13,13;28,20)
Falta tempo para levar à unidade básica de saúde	61,6 (53,0;69,7)	1,09 (1,03;1,16)	8,79 (3,57;14,08)
Não tem dinheiro para o transporte	69,3 (56,2;80,0)	1,69 (1,56;1,82)	40,91 (36,22;45,25)
Não sabe quando deve levar à unidade básica de saúde	67,0 (54,2;77,7)	1,19 (1,09;1,30)	16,33 (8,94;23,12)
Foi à unidade básica de saúde, mas não conseguiu vacinar	55,8 (53,2;58,4)	0,99 (0,97;1,01)	0,39 (0,00;2,35)
Tempo de espera longo	53,9 (46,7;61,0)	1,07 (1,01;1,12)	6,56 (1,88;11,01)
Faltou algum dos documentos solicitados na unidade básica de saúde	59,3 (49,9;68,2)	1,15 (1,09;1,23)	13,75 (8,32;18,86)
O profissional não recomendou tomar várias vacinas no mesmo dia	60,4 (54,5;66,2)	1,07 (1,02;1,12)	6,75 (2,33;10,97)

Para os motivos associados a oportunidades perdidas, o mais relevante foi a falta de documento, responsável por 13% de redução na cobertura. Chama atenção o fato de profissionais das salas de vacina não recomendarem a aplicação simultânea de vacinas e o problema com as filas.

## DISCUSSÃO

A hesitação vacinal é um fenômeno complexo que abarca as percepções sobre vacinas e as intenções dos pais. No estudo realizado em 39 cidades brasileiras, incluindo todas as regiões, foram encontradas proporções altas de hesitação vacinal em todas as camadas sociais. Tal hesitação é mais aparente nos estratos socioeconômicos mais baixos, em famílias com menor escolaridade e renda, como no Canadá.^13^

Os dados mostraram importância das questões de acesso, suplantando hesitação como motivo de menor cobertura vacinal. As questões programáticas apareceram como motivos para oportunidades perdidas, embora aparentemente com menor impacto sobre a cobertura vacinal. 

O inquérito sobre percepção da ciência e da saúde conduzido pelo Welcome Trust em 140 países, em 2016, mostrou que 79% das pessoas concordam que as vacinas são seguras, com grande variação entre as regiões do mundo. Na América Latina, 63% concordam que as vacinas são seguras, 82% que elas são efetivas e 97% acreditam que as vacinas são importantes para a saúde.^14^

Inquérito realizado em 67 países, em 2016, para avaliar a confiança nas vacinas, encontrou grande variabilidade nas respostas a questões relativas à importância, segurança e efetividade. Homens deram menos importância às vacinas, e adultos e idosos reconheceram mais a efetividade. Não houve diferenças de escolaridade e renda, mas sentimentos negativos quanto à segurança foram mais comuns em países ricos, com maior produto interno bruto *per capita*, maior gasto em saúde e maior nível de escolaridade. ^15^

Os estudos empíricos sobre hesitação têm mostrado que o fenômeno é contexto específico, não havendo preditores que se observem em todas as situações.^16^ Os dados deste estudo mostram menor hesitação nos estratos sociais mais altos, com maior escolaridade e maior renda diferindo de estudos anteriores realizados no país e em cidades como São Paulo. ^17,18^

Glassman e Szymczak relatam diferenças entre a classe média e a classe trabalhadora nos Estados Unidos, em 2018^19^ mostram que em famílias de classe média, brancas, com renda anual maior de US$ 70 mil e com ensino superior, é mais frequente a recusa a todas ou a algumas vacinas. As famílias da classe trabalhadora, com pais não anglo-saxões, sem escolaridade superior e abaixo da linha de pobreza, têm mais crianças com esquema incompleto por razões associadas a barreiras de acesso, custo, questões logísticas e menor cobertura por seguro. 

Há um amplo espectro de atitudes sobre vacinas incluindo aqueles que são favoráveis às vacinas e aceitam todas elas, aqueles que têm muitas dúvidas, mas vacinam total ou parcialmente seus filhos, e aqueles poucos que recusam totalmente as vacinas.^20^ Este estudo corrobora essas observações mostrando que, mesmo entre aqueles que declararam que não pretendiam vacinar seus filhos, há crianças com o esquema vacinal completo. A hesitação vacinal, expressa pela decisão de vacinar total ou parcialmente ou não vacinar, prejudica o alcance das coberturas almejadas. As barreiras de acesso e dificuldades com o programa desenham um quadro complexo de circunstâncias que contribuem para as quedas da cobertura em praticamente todos os países.

Diferentes modelos conceituais têm sido propostos para compreender os componentes do processo de tomada de decisão pelos pais. Dubé e colaboradores (2013) propuseram conjunto amplo de determinantes agrupados em: contexto histórico, político e sociocultural, políticas de vacinação, comunicação e mídia e profissionais de saúde.^3^ Em 2018, esses autores formularam um modelo ecológico-social rearranjando os determinantes segundo níveis hierárquicos: política de vacinação (oferta, custos, calendário), comunidade (normas sociais, perspectivas socioculturais sobre saúde e vacinas), nível organizacional (serviços, provedores e profissionais de saúde), nível interpessoal (família, amigos, redes sociais e estilos de vida) e nível individual (conhecimentos, crenças, atitudes, valores e características sociodemográficas).^16,21^

Fatores sociais como os conhecimentos, as experiências passadas, a percepção da importância das vacinas, a percepção do risco e a confiança, as normas subjetivas e as convicções religiosas influenciam a decisão da vacinação.^3,22^ Ver a vacinação como norma social é um determinante importante de aceitação assim como a responsabilidade social.^22^ O contexto pós-moderno de questionamento da validade da ciência, da competência e das autoridades médicas e sanitárias favorece os questionamentos e o ceticismo.^16^ Este estudo indica que as pessoas que não acreditam que vacinar sua criança seja importante para garantir a saúde de outras crianças no bairro têm maior probabilidade de ter esquemas incompletos.

Contraditoriamente, o foco da promoção de saúde centrado nos estilos de vida saudável e nas ações individuais, bem como o incentivo à participação ativa dos indivíduos nas decisões sobre sua saúde e a de seus filhos, contribuem para as dúvidas sobre importância e necessidade das vacinas, assim como a adesão a diferentes práticas complementares ou alternativas.^3,9,23,24^ Muitos pais acreditam que é preferível que as crianças tenham a doença, pois assim teriam uma proteção duradoura sem sobrecarregar o sistema imunológico. Embora tendam a considerar a importância das vacinas de uma perspectiva mais genérica, não acreditam que ela seja necessária para as suas crianças, visto que estariam protegidas por um estilo de vida saudável.^9,24,25^

Dimensões da percepção do risco, tais como vulnerabilidade e gravidade das consequências caso o dano ocorra, podem pender para a vacinação e para a omissão, levando ao atraso, à escolha de vacinas ou até mesmo à recusa. Na medida em que os riscos representados pelos efeitos adversos são imediatamente detectáveis nos indivíduos e os benefícios são mais difíceis de observar individualmente, a percepção do risco pode pender para a hesitação mais do que para a aceitação. O uso de dados dos sistemas de notificação de efeitos adversos sem o devido cuidado de verificar a comprovação do nexo causal é muitas vezes utilizado para alimentar as falsas notícias e a preocupação com a segurança das vacinas.^2,3,^ ^24,25,26,27,28^

Falta de conhecimento sobre quem, onde e quando vacinar também aparece entre as razões para não ter o esquema completo, tanto neste estudo como em outros. A acessibilidade e a conveniência favorecem a aceitação, porém experiências negativas com os serviços aumentam a rejeição.^3,25^ O aumento do número de vacinas e os diferentes esquemas utilizados podem aumentar a percepção negativa sobre a necessidade e relevância das vacinas.^3,^ ^24,25^

Os profissionais de saúde são as principais fontes de orientação sobre vacinas para os pais. As atitudes positivas com relação às vacinas influenciam a aceitação pelos pais.^29,30^ É muito importante que eles estejam adequadamente preparados para discutir as dúvidas e orientar de maneira segura. Muitos profissionais têm pouco conhecimento sobre vacinas, calendário, importância e veracidade dos efeitos adversos.^3,27^ Este estudo aponta que a orientação de profissionais das salas de vacina em desacordo com o calendário proposto, evitando a administração simultânea de várias vacinas, está associado à maior probabilidade de esquemas incompletos. Isso aumenta o número de visitas necessárias para o cumprimento do calendário e pode gerar doses inválidas ao desrespeitar a oportunidade e o intervalo correto entre doses. 

As limitações do estudo estão relacionadas à dificuldade inerente aos procedimentos quantitativos quando se pretende compreender fenômenos sociais complexos. Logra-se quantificar as estratificações, mas muitas vezes não se entendem as aparentes contradições. 

A intenção de não vacinar se mostrou associada à cobertura incompleta aos 24 meses, bem como a falta de confiança e as percepções de que as vacinas fazem mal à saúde, não são importantes nem para a criança em particular, nem para as demais crianças do bairro. Os problemas de acesso são mais importantes como motivos para a baixa cobertura do que as questões de hesitação vacinal, destacando-se falta de tempo e de dinheiro, perda da caderneta e desconhecimento sobre quando deve levar a criança para vacinar. Problemas de desempenho do programa também acabam contribuindo para menores coberturas, destacando-se as filas, a falta de documentos e as recomendações dos profissionais das salas de vacina para não aplicar simultaneamente todas as doses estabelecidas pelo calendário vacinal.
